# Alcohol and Cirrhosis

**Published:** 1898-01-01

**Authors:** 


### Alcohol and Cirrhosis.

The "gin-drinker's liver," its mode of occurrence, its common causation by the use of ardent spirits and morning nips, and the miserable consequences it produces—the dyspepsia, the emaciation, the hæmatemasis, and the dropsy, are all texts on which many a sermon might be preached.

Yet it must be confessed that, as in so many other sermons, there is a weak spot—a little gap, a want of continuity between the text and the discourse, which we would gladly see bridged over. That cirrhosis occurs as a consequence not of spirit drinking alone, but of many forms of alcoholic excess, is beyond all question. But what are the steps by which the evil consequence is produced seems to be involved in very considerable obscurity.

The doubts which hang over this question are well illustrated by the facts and theories which have lately been brought before the Paris Académie de Médicine by Professor Lancereaux; for when we find so careful a pathologist and so acute an observer maintaining that the cirrhosis which occurs among chronic drinkers is caused by certain salts which occur in the wines consumed, rather than by the alcohol which they contain, we may fairly admit that all cannot be quite plain sailing in the etiology of the disease.

If, instead of studying the developed disease, we take the other end of the question, and study the world, and the doings thereof, and the way men live, we soon see clearly enough that plenty of people soak themselves in alcohol and yet do not develop cirrhosis. As is said in Allbutt's "System of Medicine," "the degree of cirrhosis has no constant relation to the total amount of alcohol taken. A history of great habitual alcoholic excess may sometimes be obtained from patients who present no evidence of cirrhosis." An extreme example of this kind came to light at an inquest held last week, at Bethnal Green, on a man aged fifty-four. According to the account in the daily papers, he had long been subject to sickness and pains in the stomach, the results of his chewing large quantities of tobacco. In regard to his drinking habits, the following curious evidence was given by the widow: "The Coroner: Did he drink? Witness: I should think he did. The Coroner: What did he drink? Witness: Whisky in the evening. The Coroner: And what during the day? Witness: Old ale and stout. The Coroner: How much whisky did he drink? Witness: Eighteen half-quarterns a night. The Coroner: Do you mean every night? Witness: Yes, that was his usual. He drank that mostly after he had done his work. The Coroner: And how much ale and stout did he drink? Witness: I don't know what he had at work, but in an evening he would drink three or four half-pints at a time. The Coroner: He must have been a most remarkable man. How long has he been drinking eighteen half-quarterns a night? Witness: Oh, for the last fifteen years. He would have it. The Coroner: No wonder he complained of pains in the stomach. Could he eat his meals? Witness: Yes; after he'd had his fill of whisky he could eat better than before he had it." The medical evidence was to the effect that the body was badly nourished. "The stomach was inflamed and ulcerated, and produced peritonitis. There was evidence of chronic pneumonia and pleurisy, but otherwise the organs were healthy. The Coroner (surprised): What, the liver and kidneys healthy? Witness: Yes." It is, in fact, one of the surprises in a doctor's life to see how long some great topers live, and how comparatively comfortably they get on, notwithstanding the enormous amount of alcohol they imbibe. It is quite clear, from the observations of Professor Lancereaux and many others, that the gin-drinking hypothesis—that is, the idea that strong spirits are necessary for the production of cirrhosis, must be given up, for in a large proportion of his cases wine alone was the form in which alcohol was taken. But our English experience, on the other hand, in regard to the action of gin pure and simple, ought to be sufficient to make it quite clear that it is equally an error to attribute the mischief to the salts contained in certain forms of wine. The only link between the wine, the whisky, and the gin seems to be the alcohol which they all contain, but how the alcohol produces the cirrhosis is by no means obvious.

The special implication of the liver, which is the first organ the alcohol reaches, is a capital bit of crude pathology for the teetotal platform, but what we now know, together with the somewhat important fact that the cirrhotic tissue is not fed by the blood which comes from the intestines, but derives its nourishment from the general circulation, is enough to show that although *ben trovato* it is not true. Perhaps if we knew the real truth, it would turn out that some people digest their alcohol and others don't. But that is another story.

